# Metabolism, health and fillet nutritional quality in Atlantic salmon (*Salmo salar*) fed diets containing *n*-3-rich microalgae

**DOI:** 10.1017/jns.2015.14

**Published:** 2015-06-11

**Authors:** Katerina Kousoulaki, Tone-Kari Knutsdatter Østbye, Aleksei Krasnov, Jacob Seilø Torgersen, Turid Mørkøre, John Sweetman

**Affiliations:** 1 Nofima AS, Department of Nutrition and Feed Technology, N-5141 Fyllingsdalen, Norway; 2 Alltech Inc., Sarney, Dunboyne, County Meath, Republic of Ireland

**Keywords:** *n*-3 Long-chain PUFA, Farmed salmon fish fillet nutritional quality, Microalgae, 0_ScYE, 0 g/kg *Scizochytrium* sp. + yeast extract (control), 1_ScYE, 10 g/kg *Scizochytrium* sp. + yeast extract, 6_ScYE, 60 g/kg *Scizochytrium* sp. + yeast extract, 15_ScYE, 150 g/kg *Scizochytrium* sp. + yeast extract, ADC, apparent digestibility coefficient, CK, creatine kinase, FAME, fatty acid methyl esters, FCR, feed conversion ratio, iNOS, inducible nitric oxide synthase, ISO, International Organization for Standardization, *n*-3 LC-PUFA, *n*-3 long-chain PUFA, ScYE, *Scizochytrium* sp. + yeast extract, TGC, thermal growth coefficient

## Abstract

Microalgae, as primary producers of EPA and DHA, are among the most prominent alternative sources to fish oil for *n*-3 long-chain PUFA in animal and human nutrition. The present study aimed to assess technical, nutritional and fish health aspects of producing *n*-3-rich Atlantic salmon (*Salmo salar*) fish fillets by dietary supplementation of increasing levels of a DHA-producing *Schizochytrium* sp. and reduced or without use of supplemental fish oil. Atlantic salmon smolt were fed diets with graded levels of microalgae for 12 weeks, during which all fish showed high feed intake rates with postprandial plasma leptin levels inversely correlating with final mean fish body weights. Fish performance was optimal in all experimental treatments (thermal growth coefficient about 4·0 and feed conversion ratio 0·8–0·9), protein digestibility was equal in all diets, whereas dietary lipid digestibility inversely correlated with the dietary levels of the SFA 16 : 0. Fillet quality was good and similar to the control in all treatments in terms of *n*-3 long-chain PUFA content, gaping, texture and liquid losses during thawing. Histological fluorescence staining and immunofluorescence analysis of salmon intestines (midgut: base of intestine and villi) revealed significant effects on slime, goblet cell production and inducible nitric oxide synthase (iNOS) activity with increasing levels of dietary *Schizochytrium* sp. supplementation. Microarray analysis did not reveal any signs of toxicity, stress, inflammation or any other negative effects from *Schizochytrium* sp. supplementation in diets for Atlantic salmon.

A large body of literature associates the consumption of *n*-3 long-chain PUFA (*n*-3 LC-PUFA), in particular EPA and DHA, with multiple benefits to human health, related to reduction of CHD-associated risk factors and mortalities^(^^1^^,^^2^^)^, IHD mortality and stroke^(^^3^^)^. The suggested mechanisms responsible include the hypotensive^(^^4^^)^, hypotriacylglycerolaemic^(^^5^^)^, anti-arrhythmic^(^^6^^)^, anti-thrombotic^(^^7^^)^, anti-atherogenic and anti-inflammatory^(^^8^^,^^9^^)^ properties of *n*-3 LC-PUFA. Besides CHD, fish and fish oils may act preventively against several other diseases such as rheumatoid arthritis^(^^10^^)^, depression^(^^11^^)^, cognitive decline^(^^12^^)^, neurological disorders, such as Alzheimer's disease^(^^13^^)^, and psoriasis^(^^14^^)^. The main readily available food source of *n*-3 LC-PUFA is fish and seafood^(^^15^^)^ as well as commercially available *n*-3 LC-PUFA nutrition additives; however, health benefits are associated with the consumption of either one of them^(^^16^^–^^20^^)^. Food-grade fisheries providing fish oil and fishmeal may have already reached their limit of sustainability^(^^21^^–^^27^^)^; thus further growth in the world supply of fish, fish oil and crustaceans can only be supported based on increased aquaculture activity. Global fish oil production is approximately one million tons annually, and the salmon feed industry alone utilises about 50 % of this^(^^28^^,^^29^^)^. Further sustainable aquaculture growth with lower environmental impacts depends on increasing supplies of alternative marine *n*-3 LC-PUFA-rich feed ingredients to maintain good fish health and welfare standards and high consumer product quality^(^^30^^)^.

Microalgae are recognised as among the most prominent future sustainable sources of *n*-3 LC-PUFA-rich oils^(^^31^^,^^32^^)^. Mainly phototrophic^(^^33^^,^^34^^)^, but recently also some heterotrophic^(^^35^^,^^36^^)^, microalgae products have been tested in aqua feeds with variable performance results. Besides *n*-3 LC-PUFA, microalgae also contain, as other unicellular organisms, bioactive cell wall material compounds such as β-glucans, and a multitude of other bioactive components, as for instance, β-carotenes, flavonoids, nucleotides and water-soluble peptides. Those may affect nutrient availability and growth performance, but also enhance the well-being of fish by improving gut health and thus nutrient assimilation and immune competence and thus resistance to pathogens and disease^(^^37^^–^^43^^)^. Nevertheless, there are still concerns surrounding the viability of using single cell oils in aquaculture, associated with high production costs, high technological development requirement, and limited production capacity of mainly phototrophic microalgae^(^^44^^,^^45^^)^. In that sense, microalgae are less probable to be used in the near future as a protein source and fishmeal alternative in aqua feeds, as long as other available, far more economic, efficient marine and plant-based high-protein raw materials exist^(^^46^^–^^48^^)^. Nevertheless, large-scale production of heterotrophic microalgae by established fermentation technology, such as *Schizochytrium* sp., with high lipid content (55–75 % in DM) and up to 49 % DHA of total lipids is practised today^(^^49^^)^. Such products can in the near future target the cost-efficient supplementation of aqua feeds with *n*-3 LC-PUFA to meet the nutritional requirements of farmed organisms and maintain the health properties of aquaculture products for human consumption.

To date, there is very limited available information on the nutritional quality, digestibility and functional effects of feeding high dietary levels of whole heterotrophic microalgae biomass to Atlantic salmon (*Salmo salar*). The main objective of the present study was to enrich the existing knowledge on the feasibility to ease the pressure on fish oil as a source of *n*-3 LC-PUFA in Atlantic salmon feeds using DHA-rich *Schizochytrium* sp. microalgae, maintaining fish health, performance and adequate *n*-3 LC-PUFA level in the fillets. Results on *n*-3 LC-PUFA and SFA levels in salmon fillets, fillet quality parameters: lipid-holding capacity, lipid fat losses during storage and fillet gaping, and Atlantic salmon production performance, gut health and global gene expression are presented.

## Materials and methods

### Raw materials, diets and fish experiment

Four commercially relevant^(^^50^^)^ Atlantic salmon diet formulations were created (Table 1) with increasing levels (up to 150 g/kg) spray-dried algae (*Schizochytrium* sp.) biomass adjusted to 50 % crude lipid content by supplementation of yeast extract (Alltech Inc.) and decreasing levels of supplemental fish oil (down to 0 g/kg). The dietary inclusion levels of ScYE (*Scizochytrium* sp. + yeast extract) tested were 0, 10, 60 and 150 g/kg. The dietary formulations were balanced on the basis of calculated digestible protein, energy and amino acids, soluble P and total EPA + DHA. The experimental diets were produced by extrusion in the same production series, under standard setup conditions at the Feed Technology Center of Nofima in Titlestad, Norway.

**Table 1.** Dietary formulations in Atlantic salmon (*Salmo salar*) feeding experiment (raw material content is given in g/kg raw material mix)

Diet…	Diet 1, 0_ScYE	Diet 2, 1_ScYE	Diet 3, 6_ScYE	Diet 4, 15_ScYE
Fishmeal	248	248		248
Soya protein concentrate	180	180	180	180
Maize gluten	119	119	117	43
Wheat gluten	60	59·5	58	100
Horse beans	91·8	86·8	66·7	54·6
Soya lecithin	5	5	5	5
Rapeseed oil	75	75	75	152
Fish oil	153	148·5	122·4	
ScYE*	–	10	60	150
Inositol	0·3	0·3	0·3	0·3
Betafin	5	5	5	5
Mineral premix – P†	5·2	5·2	5·2	5·2
NaH_2_PO_4_	26·3	26·3	26·0	25·6
Vitamin mix†	20	20	20	20
Lysine	2·5	2·5	2·5	2·5
Methionine	7·9	7·9	7·9	7·9
Astaxanthine (10 %)	0·5	0·5	0·5	0·5
Yttrium oxide	0·5	0·5	0·5	0·5
Sum	1000	1000	1000	1000

0_ScYE, 0 g/kg *Scizochytrium* sp. + yeast extract (control); 1_ScYE, 10 g/kg *Scizochytrium* sp. + yeast extract; 6_ScYE, 60 g/kg *Scizochytrium* sp. + yeast extract; 15_ScYE, 150 g/kg *Scizochytrium* sp. + yeast extract.

* Heterotrophically produced *Scizochytrium* sp., spray dried and balanced to 500 g/kg total fat by yeast extract (Alltech Inc.).

† Providing in the final diet: 3000 IU vitamin D_3_, 160 mg/kg vitamin E, 20 mg/kg vitamin K_3_, 200 mg/kg vitamin C, 20 mg/kg vitamin B_1_, 30 mg/kg vitamin B_2_, 25 mg/kg vitamin B_6_, 0·05 mg/kg vitamin B_12_, 60 mg/kg vitamin B_5_, 10 mg/kg folic acid, 200 mg/kg vitamin B_3_, 1 mg/kg vitamin B_7_, 92·31 mg/kg MnSO_4_ + H_2_O, 3125 mg/kg MgHPO_4_ + 3H_2_O, 182·4 mg/kg FeSO_4_ + H_2_O, 352·9 mg/kg ZnSO_4_ + H_2_O, 23·62 mg/kg CuSO_4_ + H_2_O, 1413 mg/kg K_2_CO_3_ and 6·67 mg/kg Se.

Unfed (24 h) Atlantic salmon smolts from Sunndalsøra, Norway, of 213 g initial body weight, were used for the current trial which lasted for 12 weeks. Groups of between ten and twenty fish were bulk weighed and transferred to each one of twelve experimental tanks to end up with forty fish per tank (8 kg/m^3^ fish stocking density). The mean water temperature during the trial was 10·2 °C, water flow was continuous (30 litres/min, 180 %/h), and water salinity ranged between 32 and 33 parts per thousand. The water system was flow-through using UV-treated filtrated seawater from 40 m depth. The fish were first fed the day following the start of the trial, in gradually increasing amounts in order to determine the satiation feeding levels of each group. Fish were fed continuously in satiation (115·6 % of *ad libitum* levels) using automatic feeders until the moment of sampling for each individual tank. Uneaten pellets were collected daily to monitor the daily feed intake of each experimental population. Five fish per tank were randomly removed at the trial start and also after 6 weeks for total lipid and fatty acid analysis in Norwegian quality cut fillet samples. At the trial end, five non-stripped randomly selected fish, excluding too big, too small fish or fish with empty stomachs, were used for the sampling of blood, Norwegian quality cut fillet and internal organs (liver and intestine) for further chemical analyses and biological studies. All other fish from each tank were stripped and their faeces separated from urine, collected in a pre-weighed box per tank, and frozen immediately at –20 °C prior to further analyses.

### Chemical composition analyses

Crude protein in diets and faeces was determined by the Kjeldahl method (N × 6·25) (ISO (International Organization for Standardization) 5983–1997). Moisture (ISO 6496–1999) and ash (ISO 5984–2002) were determined gravimetrically after drying pre-weighed samples in porcelain cups at 105 °C for 24 h and then incinerating the dried samples at 500 °C for 12 h, respectively. Total lipid in raw materials, diets and the body tissues was quantified by the Bligh & Dyer extraction method^(^^51^^)^. Dietary gross energy was determined in a Parr adiabatic bomb calorimeter. For total amino acid profile determination, samples were hydrolysed in 6 m-HCl for 22 h at 110 °C and analysed by HPLC using a fluorescence technique for detection^(^^52^^)^. Total P was determined by a spectrophotometric method (ISO 6491–1998). The water-soluble fraction of the diets was extracted with boiling water. The extract was then filtered using paper filter, and the crude protein content in the water phase was determined by the Kjeldahl method. Analysis of fatty acid composition was realised in Bligh & Dyer^(^^51^^)^ extracts. Preparation of fatty acid methyl esters (FAME) was done according to AOCS Official Method Ce 1b-89. The GC analyses was conducted on a Trace GC gas chromatograph (Thermo Fisher Scientific) with a flame ionisation detector (GC–FID), equipped with a 60 m × 0·25 mm BPX-70 cyanopropyl column with 0·25 μm film thickness (SGE Analytical Science). He 4·6 was used as the mobile phase under the pressure of 2·60 bar. The injector temperature was 250 °C and the detector temperature was 260 °C. The oven was programmed as follows: 60 °C for 4 min, 30 °C/min to 164 °C, and then 1·0 °C/min to 213 °C, and 100 °C/min to 250 °C where the temperature was held for 10 min. The sample solution (3·0 μl) was injected splitless and the split was opened after 2 min. The FAME were identified by comparing the elution pattern and relative retention time with the reference FAME mixture (GLC-793; Nu-Chek Prep Inc.). Chromatographic peak areas were corrected by empirical response factors calculated from the areas of the GLC-793 mixture. Fatty acid composition was calculated by using 23 : 0 FAME as the internal standard and reported on a sample basis as g/100 g FAME. All analyses were performed in duplicate. If differences between parallels exceeded standardised values, new duplicate analyses were carried out according to accredited procedures (Table 2).

**Table 2.** Experimental raw material and diet chemical composition, given in g/kg diet as-is or as-fed, respectively, for proximate composition and dietary ScYE* content, in kJ/g diet as-fed for dietary crude energy content, and in g/kg Bligh and Dyer extract for fatty acid (FA) and lipid class composition

	ScYE	Diet 1, 0_ScYE	Diet 2, 1_ScYE	Diet 3, 6_ScYE	Diet 4, 15_ScYE
Supplemental dietary fish oil		153	148·5	122·4	0
Dietary ScYE		0	10	60	150
Proximate composition					
Crude protein	178	443	437	425	445
Moisture	27	81	78	73	77
Crude ash	37	74	75	73	76
Total lipid	544	272	281	305	278
Gross energy		227·1	229·4	236·6	230·3
Lipid fatty acids					
Sum SFA	577	163	167	212	244
Sum MUFA	4	468	452	448	378
Sum PUFA (*n*-6) FA	7	91	88	90	120
Sum PUFA (*n*-3) FA	264	167	165	181	147
Sum PUFA FA	271	263	258	275	267
Sum FA	852	894	877	935	889
14 : 0	44	39	38	39	20
16 : 0	515	104	109	151	202
18 : 0	15	17	17	18	16
20 : 0	2	2	02	3	04
22 : 0	1	1	01	1	2
16 : 1*n*-7	1	32	30	28	7
18 : 1(*n*-9) + (*n*-7) + (*n*-5)	3	263	253	265	335
20 : 1(*n*-9) + (*n*-7)	<1	74	72	67	18
22 : 1(*n*-11) + (*n*-9) + (*n*-7)	<1	95	93	85	17
24 : 1*n*-9	<1	4	4	3	1
16 : 2*n*-4	<1	3	3	2	<1
16 : 3*n*-4	<1	2	2	2	<1
18 : 2*n*-6	6	88	85	88	120
18 : 3*n*-6	<1	<1	<1	<1	<1
20 : 2*n*-6	<1	1	1	1	<1
20 : 3*n*-6	1	<1	<1	<1	<1
20 : 4*n*-6	<1	2	2	1	<1
22 : 4*n*-6	<1	<1	<1	<1	<1
18 : 3*n*-3	<1	29	28	30	40
18 : 4*n*-3	<1	18	17	16	3
20 : 3*n*-3	<1	1	<1	1	<1
20 : 4*n*-3	4	4	4	4	1
20 : 5*n*-3 (EPA)	2	45	43	40	11
21 : 5*n*-3	<1	2	2	2	<1
22 : 5*n*-3	<1	5	5	4	1
22 : 6*n*-3 (DHA)	258	63	66	84	91
Lipid classes					
TAG	870				
Diacylglycerol	<5				
Monoacylglycerol	<10				
NEFA	28				
Sterols	14				
Total polar lipids	<10				

0_ScYE, 0 g/kg *Scizochytrium* sp. + yeast extract (control); 1_ScYE, 10 g/kg *Scizochytrium* sp. + yeast extract; 6_ScYE, 60 g/kg *Scizochytrium* sp. + yeast extract; 15_ScYE, 150 g/kg *Scizochytrium* sp. + yeast extract.

* Heterotrophically produced *Scizochytrium* sp., spray dried and balanced to 500 g/kg total fat by yeast extract (Alltech Inc.).

Salmon plasma leptin hormone levels were measured using the Fish (salmon) leptin (LEP) ELISA kit (Cusabio) following the manufacturer's protocol. Absorbance was measured at 450 nm in a Spectrostar Nano microplate reader from BMG LabTech GmbH. Plasma creatine kinase (CK) is a measure of muscle degradation. As an indicator of muscle damage, enzymic activity of CK was analysed spectrophotometrically in salmon plasma (Avida^®^1650 analytical instrument; Bayer)^(^^53^^)^.

The apparent digestibility coefficient (ADC) of nutrients and energy in the test diets was calculated from the following formula: ADC = 100–100 × Yd × Nf / Nd / Yf, were d is diet, f is faeces, Y is yttrium content and N is nutrient content. Thermal growth coefficient (TGC) was calculated from the following formula: TGC = (w_2_^1/3^–w_1_^1/3^) × 1000/∑(t × feeding days), where ∑(t × feeding days) is the sum of water temperatures (°C) for every feeding day in the experiment^(^^54^^)^. Feed intake was calculated as the mean feed consumption per fish per day as % of the daily fish body weight. The daily fish body weight was calculated using daily TGC values equal to the overall TGC of each tank. Feed conversion ratio (FCR) is feed consumed/biomass increase. Protein efficiency ratio is fish weight gain/protein consumption. Condition factor is (fish weight (g)/fish fork length^3^) × 1000. Dress-out percentage is (gutted fish weight/whole fish weight) × 100. Hepatosomatic index (HSI) is 100 × (liver weight/whole fish weight).

### Fish fillet technical quality

Fish fillet technical quality was evaluated in terms of fatty acid composition, liquid-holding capacity, gaping and texture. Liquid-holding capacity was analysed as the ability of a standardised muscle sample (15 g) to retain water and fat, respectively, during storage and was analysed as weight decreased during thawing the muscle (75 g) at 20 °C for 20 h^(^^55^^)^. Fillet gaping was analysed according to a visual scale ranging from 0 to 5, where 0 corresponds to no gaping and score 5 extreme gaping^(^^56^^)^. Instrumental analyses of salmon fillets were performed using a Texture analyser, TA-XT2 (Stable Micro System Ltd) equipped with a flat-ended cylindrical probe (12·5 mm diameter, type p/0.5) and a 30 kg load cell. Firmness predicted using this method correlates well with sensory assessment of firmness of raw and smoked salmon fillets^(^^57^^)^.

### Microscopic analysis of intestinal tissue

For microscopic analysis, intestinal tissue was sampled just aft of the pyloric caeca. After removal of the intestinal content by rinsing with 1 × PBS, fixation was achieved in 4 % paraformaldehyde (PFA). Sampled tissue was dehydrated and embedded in polyester wax and sectioned at 7 μm^(^^58^^)^. Intestinal morphology was assessed by Phalloidin and wheat germ agglutinin (WGA) staining, which visualises F-actin in the tight junctions and connective tissue/mucus, respectively. Samples were also analysed by immunofluorescence to assess the effect of dietary level of ScYE upon oxidative stress using an antibody against inducible nitric oxide synthase (iNOS)^(^^59^^)^. This protein is a key player in the intestinal immune system and controls inflammatory cytokines such as TNFα. As ScYE was a new raw material for salmon nutrition we were unaware of the anticipated effects and the scope of the microscopic analyses was to obtain indications of possible effects. Thus only five fish per dietary treatment were analysed.

### Microarray analyses

RNA was extracted using PureLink RNA Mini kits according to the manufacturer's protocol (Invitrogen). Concentration of total RNA (NanoDrop 1000 Spectrometer; Thermo Scientific) and RNA integrity were measured (Agilent 2100 Bioanalyser with RNA Nano kits; Agilent Technologies). Samples with RNA integrity number (RIN) > 8 were accepted for analyses. Multiple gene expression profiling was performed with Nofima's Atlantic salmon 15 k oligonucleotide microarray SIQ6 (GEO Omnibus GPL16555) produced by Agilent Technologies. Two-colour hybridisations were performed. Analyses included four replicates from each study group; in total sixteen microarrays were used. RNA amplification, labelling and fragmentation were performed using the two-colour low-input quick AMP labelling kit and gene expression Hybridisation kit following the manufacturer's instructions (Agilent Technologies). The input of total RNA in each reaction was 100 ng. Test samples and reference (equalised mixture of all samples) were labelled with Cy5 and Cy3 (cyanine) , respectively. Overnight hybridisation (17 h; 65 °C; rotation speed of 10 rpm) was executed in an oven (Agilent Technologies). The slides were washed with Gene Expression Wash Buffers 1 and 2 and scanned with a GenePix 4100A (Molecular Devices) at 5 μm resolution. The GenePix Pro software (version 6.1) was used for spot-to-grid alignment, feature extraction and quantification. Assessment of spot quality was done with GenePix flags. Nofima's bioinformatics package STARS^(^^60^^)^ was used for data processing and mining. After filtration of low-quality spots, LOWESS (locally weighted scatterplot smoothing ) normalisation of log_2_-expression ratios (ER) was done. The differentially expressed genes were selected by criteria by difference from the control: log_2_-ER > |0·8| (1·75-fold) and *P* < 0·05 (online Supplementary Table S1).

### Statistics

Data were tested for normality using a Kolomogorov–Smirnov test and homogeneity of variance using Levene's test, and when necessary, transformed via arcsine function. One-way ANOVA was used for the statistical analyses of the whole dataset, whereas two-way ANOVA and correlation statistical analyses were performed in addition of the fillet fatty acid composition data to test effects of time and diet. The statistical analyses presented were performed using Microsoft Excel and SPSS 10.0 for Windows. When significant differences among groups were identified, multiple comparisons among means were made using the Tukey test. Differences were considered significant at the level of *P* < 0·05.

## Results

### General fish performance

Fish survival, feed intake, feed conversion and protein efficiency rates were similar among the different treatments (Table 3). Fish fed 1 % ScYE in the diet had the highest body growth rate significantly only when compared with fish fed in treatment 15_ScYE. There was a significant positive correlation between feed intake and FCR (*R*^2^ 0·79; *n* 12), but not between feed intake and growth. There were no significant effects of ScYE dietary incorporation on liver size (HSI) or fish condition factor. Fish fed the diet 15_ScYE had significantly higher fillet yield (dress-out percentage) compared with the other treatments. Significantly reduced postprandial leptin levels were analysed in the blood plasma of the fish in treatment 1_ScYE compared with the other three treatments.

**Table 3.** Performance, biometrics and postprandial plasma leptin of Atlantic salmon (*Salmo salar*) fed diets with increasing levels of ScYE* and decreasing levels of fish oil (Mean values with their standard errors; *n* 3, except for plasma leptin: *n* 15)

Treatment…	0_ScYE	1_ScYE	6_ScYE	15_ScYE	sem
Supplemental dietary fish oil (g/kg diet)	153	148·5	122·4	0	
Dietary ScYE (g/kg diet)	0	10	60	150	
Start fish number	40	40	40	40	0
Total mortality (%)	2·5	0·0	0·8	0·8	0·6
Initial fish body weight (g)	213	213	213	213	0
Final fish body weight (g)	824^a,b^	851^b^	819^a,b^	797^a^	7
Total feed intake (kg)	18·4	21·2	21·0	19·2	0·6
FI† (% metabolic body weight/d)	1·39	1·53	1·54	1·45	0·03
FCR‡	0·78	0·83	0·88	0·83	0·02
TGC§ (× 1000)	4·03^a,b^	4·15^b^	4·01^a,b^	3·91^a^	0·03
PER||	2·89	2·76	2·71	2·73	0·07
CF¶	1·46	1·45	1·48	1·41	0·01
D%**	87·9^a^	87·8^a^	87·3^a^	88·7^b^	0·2
HSI††	1·54	1·54	1·42	1·48	0·02
Salmon postprandial plasma leptin (ng leptin/ml plasma)	63·7^b^	55·7^a^	64·4^b^	69·1^b^	1·4

0_ScYE, 0 g/kg *Scizochytrium* sp. + yeast extract (control); 1_ScYE, 10 g/kg *Scizochytrium* sp. + yeast extract; 6_ScYE, 60 g/kg *Scizochytrium* sp. + yeast extract; 15_ScYE, 150 g/kg *Scizochytrium* sp. + yeast extract; FI, feed intake; FCR, feed conversion ratio; TGC, thermal growth coefficient; PER, protein efficiency ratio; CF, condition factor; D%, dress-out percentage; HSI, hepatosomatic index.

^a,b^ Mean values within a row with unlike superscript letters were significantly different (*P* < 0·05; one-way ANOVA as separated by Tukey's *post hoc* test).

* Heterotrophically produced *Scizochytrium* sp., spray dried and balanced to 500 g/kg total fat by yeast extract (Alltech Inc.).

† FI rate is the mean feed consumption per fish per d expressed as percentage of the daily fish body weight. The daily fish body weight was calculated using daily specific growth rate values equal to the overall specific growth rate of each tank.

‡ FCR is total feed consumed/total fish biomass increase.

§ TGC = (w_2_^1/3^ – w_1_^1/3^) × 1000/ ∑(t × feeding days), where ∑(t × feeding days) is the sum of water temperatures (°C) for every feeding day in the experiment^(^^59^^)^.

|| PER is fish weight gain/protein consumption.

¶ CF = (fish weight (g)/fish fork length^3^) × 1000.

** D% = (gutted fish weight/whole fish weight) × 100.

†† HSI = % of liver weight/whole fish weight.

### Salmon fillet (Norwegian quality cut) technical quality

The technical quality of the salmon fillets at the end of the experiment was similar in all dietary treatments (Table 4). The degree of salmon fillet gaping was generally low in all experimental treatments, averaging score 1. Gaping at such a low level does not cause problems during filleting and/or processing. Instrument fillet texture analyses revealed no texture abnormalities and no significant differences were observed between dietary treatments. The results showed no significant variation in liquid loss during thawing between dietary treatments.

**Table 4.** Fillet (Norwegian quality cut) technical quality of Atlantic salmon (*Salmo salar*) fed diets with increasing levels of ScYE* and decreasing levels of fish oil (Mean values with their standard errors; *n* 3)

Treatment…	0_ScYE	1_ScYE	6_ScYE	15_ScYE	sem
Supplemental dietary fish oil (g/kg diet)	153	148·5	122·4	0	
Dietary ScYE (g/kg diet)	0	10	60	150	
Gaping score	0·7	0·9	1·1	1·1	0·2
Firmness (Newtons)	3·5	3·5	3·5	3·6	0·1
Liquid loss (%)	9·3	10·2	9·4	10·6	1·0

0_ScYE, 0 g/kg *Scizochytrium* sp. + yeast extract (control); 1_ScYE, 10 g/kg *Scizochytrium* sp. + yeast extract; 6_ScYE, 60 g/kg *Scizochytrium* sp. + yeast extract; 15_ScYE, 150 g/kg *Scizochytrium* sp. + yeast extract.

* Heterotrophically produced *Scizochytrium* sp., spray dried and balanced to 500 g/kg total fat by yeast extract (Alltech Inc.).

### Salmon fillet (Norwegian quality cut) fatty acid composition

The salmon Norwegian quality cut fillet lipid and fatty acid contents at the experiment start and after 6 and 12 weeks of feeding with the experimental diets are presented in Table 5. The total lipid levels in the fillets increased from 39 g/kg at trial start to 110–120 g/kg at trial end, and, as a consequence, the fillet levels of the different fatty acids supplemented by the diet also increased accordingly. At the end of the trial, no significant differences were observed in total fillet lipid levels among the fish fed the control and the ScYE-supplemented diets. In general terms, there was no difference between control (0_ScYE), 1_ScYE and 6_ScYE treatments, except for the higher DHA levels in treatment 6_ScYE and lower total fatty acid levels in the control fish. Fish fillets in treatment 15_ScYE had higher total *n*-6 PUFA (significantly also for 18 : 2*n*-6 and 20 : 2*n*-6), 18 : 3*n*-3 and 20 : 0 levels than the control and lower levels of EPA, 14 : 0, 16 : 1, 16 : 2*n*-4, 18 : 4*n*-3, 20 : 1, 20 : 4*n*-3, 21 : 5*n*-3, 22 : 5*n*-3, 22 : 1 and 24 : 1*n*-9 compared with all other treatments.

**Table 5.** Changes in the Norwegian quality cut fillet fatty acid content (g/kg fillet) of Atlantic salmon (*Salmo salar*) fed diets with increasing level of ScYE* and decreasing levels of fish oil after 6 and 12 weeks† (Mean values with their standard errors)

Time…	0		6 weeks					12 weeks					Two-way ANOVA (*P*)		
Treatment…	Start	sem (*n* 2)	0_ScYE	1_ScYE	6_ScYE	15_ScYE	sem (*n* 12)	0_ScYE	1_ScYE	6_ScYE	15_ScYE	sem (*n* 12)	Diet	Time	Diet x time
Total lipids (Bligh and Dyer)			88·7^b,c^	90·7^b,c^	85·7^b^	83·0^b^		117·0^c,d^	123·0^d^	122·3^d^	109·0^b,c,d^		NS	0·001	NS
	39·0^a^	2·0					2·8					2·9			
14 : 0	1·32^a^	0·03	3·02^c,d^	3·02^c,d^	2·83^b,c^	1·74^a^	0·19	3·90^c,d^	4·09^d^	3·93^d^	1·92^a,b^	0·28	0·001	0·001	NS
16 : 0	5·16^a^	0·09	10·82^b^	10·93^b,c^	11·28^b,c^	11·82^bc^	0·37	13·96^b,c,d^	14·81^c,d^	16·35^d^	16·35^d^	0·44	NS	0·001	NS
16 : 1*n*-7	1·25^a^	0·02	2·76^c,d^	2·72^c,d^	2·49^b,c^	1·44^a^	0·18	3·67^d,e^	3·77^e^	3·52^d^	1·56^a,b^	0·29	0·001	0·001	0·05
16 : 2*n*-4	0·08^a^	0·00	0·18^b,c^	0·18^b,c^	0·17^b,c^	0·08^a^	0·01	0·23^c^	0·25^c^	0·24^c^	0·12^a,b^	0·01	0·001	0·001	–
16 : 3*n*-4	0·04^a^	0·00	0·09^b,c^	0·09^b,c,d^	0·09^b^	0·08^b^	0·00	0·12^b,c,d^	0·12^d^	0·12^c,d^	0·11^b,c,d^	0·00	NS	0·001	–
18 : 0	0·97^a^	0·01	2·04^b^	2·09^b,c^	1·92^b^	1·93^b^	0·06	2·97^d^	3·03^d^	3·02^d^	2·83^c,d^	0·07	NS	0·001	NS
18 : 1(*n*-9) + (*n*-7) + (*n*-5)	9·09^a^	0·21	23·76^b^	23·82^b^	22·69^b^	26·24^b,c^	0·87	33·27^c,d^	34·51^c,d^	35·02^d^	38·76^d^	0·93	NS	0·001	0·1
18 : 2*n*-6	2·18^a^	0·07	6·61^b,c^	6·68^b,c^	6·44^b^	7·98^b,c^	0·29	8·99^c,d^	9·46^d,e^	9·76^d,e^	11·60^e^	0·35	0·001	0·01	0·05
18 : 3*n*-3	0·84^a^	0·02	2·07^b^	2·15^b^	2·12^b^	2·52^b,c^	0·09	2·73^b,c^	2·91^c,d^	3·02^c,d^	3·55^d^	0·11	0·01	0·001	0·01
18 : 3*n*-6	0·04^a^	0·00	0·09^b,c^	0·09^b,c,d^	0·09^b^	0·08^b^	0·00	0·12^b,c,d^	0·12^d^	0·12^c,d^	0·11^b,c,d^	0·00	NS	0·001	–
18 : 4*n*-3	0·45^a^	0·00	0·98^b^	0·91^b^	0·86^b^	0·44^a^	0·07	1·09^b^	1·15^b^	1·06^b^	0·44^a^	0·09	0·001	0·05	NS
20 : 0	0·06^a^	0·02	0·18^b,c^	0·18^b,c^	0·17^b^	0·17^b^	0·01	0·23^b,c^	0·25^c^	0·24^c^	0·33^d^	0·01	NS	0·001	0·01
20 : 1(*n*-9) + (*n*-7)	1·85^a^	0·07	6·07^c^	6·11^c^	5·35^b,c^	2·99^a^	0·43	8·42^d^	9·04^d^	8·25^d^	3·74^a,b^	0·66	0·001	0·001	0·1
20 : 2*n*-6	0·20^a^	0·01	0·57^b^	0·54^b^	0·51^b^	0·69^b,c^	0·03	0·82^c^	0·90^c,d^	0·86^c^	1·12^d^	0·04	0·01	0·001	0·05
20 : 3*n*-3	0·08^a^	0·00	0·18^a,b^	0·18^a,b^	0·17^a,b^	0·22^b,c^	0·01	0·23^b,c^	0·25^b,c^	0·29^b,c^	0·33^c^	0·02	NS	0·01	NS
20 : 3*n*-6	0·04^a^	0·00	0·09^a,b^	0·09^a,b^	0·09^a,b^	0·14^b,c^	0·01	0·15^b,c^	0·12^b^	0·12^b^	0·22^c^	0·01	0·01	0·01	NS
20 : 4*n*-3	0·35^a^	0·02	0·83^b^	0·82^b^	0·77^b^	0·42^a^	0·06	1·17^c^	1·23^c^	1·19^c^	0·55^a,b^	0·09	0·001	0·001	0·1
20 : 4*n*-6	0·12^a^	0·01	0·18^a,b^	0·18^b^	0·17^a,b^	0·17^a,b^	0·01	0·23^b^	0·20^b^	0·24^b^	0·22^b^	0·01	NS	0·01	NS
20 : 5*n*-3 (EPA)	1·31^a^	0·05	2·75^b^	2·69^b^	2·54^b^	1·33^a^	0·19	3·24^b^	3·36^b^	3·10^b^	1·17^a^	0·28	0·001	0·05	0·1
21 : 5*n*-3	0·08^a^	0·00	0·18^b,c^	0·18^b,c^	0·15^a,b^	0·09^a^	0·01	0·23^c^	0·25^c^	0·24^c^	−	0·00	0·05	0·001	NS
22 : 0	–	–	0·09^a^	0·09^a^	0·09^a^	0·08^a^	0·00	0·12^b^	0·12^b^	0·12^b^	0·11^b^	0·00	NS	0·001	–
22 : 1(*n*-11) + (*n*-9) + (*n*-7)	1·99^a^	0·10	6·34^b^	6·38^b,c^	5·57^b,c^	2·30^a^	0·54	8·30^c,d^	9·01^d^	8·14^c,d^	2·22^a^	0·84	0·001	0·001	0·05
22 : 5*n*-3	0·55^a^	0·03	1·06^b^	1·09^b,c^	1·00^b^	0·55^a^	0·07	1·37^b,c^	1·48^c^	1·39^b,c^	0·51^a^	0·12	0·001	0·01	–
22 : 6*n*-3 (DHA)	5·97^a^	0·02	8·98^b^	9·26^b,c^	9·68^b,c^	9·93^b,c^	0·29	9·25^b,c^	10·27^b,c^	12·20^d^	11·78^c,d^	0·40	0·05	0·01	0·01
24 : 1*n*-9	0·21^a^	0·01	0·44^b,c,d^	0·45^c,d^	0·40^b,c^	0·25^a^	0·03	0·47^c,d^	0·53^d^	0·49^c,d^	0·33^a,b^	0·02	0·001	0·01	NS
Sum SFA	7·51^a^	0·11	16·14^b,c^	16·29^b,c^	16·29^b,c^	15·74^b^	0·52	21·14^b,c,d^	22·30^d^	23·67^d^	21·54^c,d^	0·56	NS	0·001	NS
Sum MUFA	14·39^a^	0·41	39·37^b^	39·49^b^	36·50^b^	33·21^b^	1·48	54·20^c^	56·85^c^	55·42^c^	46·62^b,c^	1·62	NS	0·001	NS
Total PUFA	12·30^a^	0·20	24·84^b^	25·13^b,c^	24·84^b,c^	24·65^b^	0·78	30·01^b,c^	32·05^b,c^	33·98^c^	31·71^b,c^	0·73	NS	0·001	NS
Sum *n*-3 PUFA	9·61^a^	0·10	17·03^b,c^	17·27^b,c^	17·29^b,c^	15·45^b^	0·56	19·35^b,c,d^	20·87^c,d^	22·50^d^	18·32^b,c,d^	0·61	NS	0·01	NS
Sum *n*-6 PUFA	2·57^a^	0·09	7·53^b^	7·59^b,c^	7·30^b^	9·06^b,c,d^	0·33	10·28^c,d^	10·80^d,e^	11·11^d,e^	13·27^e^	0·40	0·01	0·001	NS
Sum fatty acids	34·21^a^	0·72	80·35^b,c^	80·90^b,c^	77·63^b^	73·61^b^	2·69	105·35^c^	111·20^d^	113·07^d^	99·87^b,c,d^	2·67	NS	0·001	0·05
EPA + DHA	7·28^a^	0·23	11·73^b^	11·95^b,c^	12·22^b,c^	11·25^b^	0·36	12·49^b,c^	13·63^b,c^	15·30^c^	12·95^b,c^	0·40	NS	0·01	0·001

0_ScYE, 0 g/kg *Scizochytrium* sp. + yeast extract (control); 1_ScYE, 10 g/kg *Scizochytrium* sp. + yeast extract; 6_ScYE, 60 g/kg *Scizochytrium* sp. + yeast extract; 15_ScYE, 150 g/kg *Scizochytrium* sp. + yeast extract.

^a–e^ Mean values within a row with unlike superscript letters were significantly different (*P* < 0·05; one-way ANOVA as separated by Tukey's *post hoc* test). *Post hoc* tests were not performed when groups had fewer than two cases.

* Heterotrophically produced *Scizochytrium* sp., spray dried and balanced to 500 g/kg total fat by yeast extract (Alltech Inc.).

† 0_ScYE: 153/0; 1_ScYE: 148·5/10; 6_ScYE: 122·4/60; 15_ScYE: 0/150 g/kg supplemental dietary fish oil/ScYE, respectively.

### Apparent digestibility coefficient of dietary energy and nutrients

Dietary protein ADC was similar between the control and all ScYE-supplemented diets in Atlantic salmon (Table 6). At the 6 and 15 % ScYE dietary supplementation levels lipid and total fatty acid ADC significantly reduced compared with the control and 1 % ScYE supplementation, mainly due to lower digestibility of all analysed SFA. In 15_ScYE, ADC of DHA (98·1 %), total *n*-3 PUFA (98·6 %) and total PUFA (98·7 %) was 1 % lower (*P* < 0·001) compared with all others treatments. The ADC of MUFA was 0·3–0·5 % higher in the ScYE supplemented fatty acids compared with the control (*P* < 0·01). Total ADC of dietary energy was highest in the 1_ScYE and lowest in the 15_ScYE treatment.

**Table 6.** Experimental diets' crude protein, crude lipid, energy and fatty acid percentage apparent digestibility coefficient (% ADC) in Atlantic salmon (*Salmo salar*) (Mean values with their standard errors)

Diet…	0_ScYE	1_ScYE	6_ScYE	15_ScYE	sem (*n* 12)
Supplemental dietary fish oil (g/kg diet)	153·0	148·5	122·4	0	
Dietary ScYE* (g/kg diet)	0	10	60	150	
ADC_protein_	87·7	87·6	87·1	87·1	0·1
ADC_lipid_	95·8^c^	96·0^c^	93·9^b^	87·8^a^	1·0
ADC_energy_	83·2^b^	84·2^c^	83·4^b^	79·7^a^	0·5
ADC14 : 0	97·2^c^	97·2^c^	92·9^b^	69·0^a^	3·5
ADC16 : 0	95·8^c^	94·4^c^	83·1^b^	64·8^a^	3·7
ADC 18 : 0	95·8^c^	95·7^c^	93·2^b^	84·7^a^	1·4
ADC 20 : 0	95·8^c^	96·0^c^	93·9^b^	90·8^a^	0·6
ADC 22 : 0	91·7^b^	92·0^b^	87·7^a^	87·8^a^	0·6
ADC 16 : 1*n*-7	99·3^b^	99·4^b^	99·3^b^	98·3^a^	0·1
ADC 18 : 1(*n*-9) + (*n*-7) + (*n*-5)	99·1	99·3	99·3	99·3	0·0
ADC 20 : 1(*n*-9) + (*n*-7)	98·3	98·7	98·8	98·4	0·1
ADC 22 : 1(*n*-11) + (*n*-9) + (*n*-7)	97·9	98·4	98·5	98·3	0·1
ADC 24 : 1*n*-9	93·8	95·0	93·9	–	0·2
ADC 16 : 2*n*-4	100	100	100	–	0·0
ADC 16 : 3*n*-4	100	100	100	–	0·0
ADC 18 : 2*n*-6	98·8	99·0	99·0	98·9	0·0
ADC 20 : 2*n*-6	98·6	100	100	–	0·4
ADC 20 : 4*n*-6	99·3	100	100	–	0·2
ADC 18 : 3*n*-3	99·4	99·6	99·6	99·6	0·0
ADC 18 : 4*n*-3	99·8	99·9	100	100	0·0
ADC 20 : 3*n*-3	100	–	100	–	0·0
ADC 20 : 4*n*-3	99·7	99·7	100	100	0·1
ADC 20 : 5*n*-3	99·5	99·6	99·6	99·3	0·1
ADC 21 : 5*n*-3	100	100	98·9	–	0·3
ADC 22 : 5*n*-3	98·9	99·5	98·9	100	0·2
ADC 22 : 6*n*-3	99·0^b^	99·1^b^	99·1^b^	98·1^a^	0·1
ADC of total SFA	96·1^c^	95·1^c^	86·0^b^	67·0^a^	3·5
ADC of total MUFA	98·7^a^	99·0^b^	99·1^b^	99·2^b^	0·1
ADC of total PUFA (*n*-6)	98·8	99·0	99·1	98·9	0·1
ADC of total PUFA (*n*-3)	99·3^b^	99·4^b^	99·4^b^	98·6^a^	0·1
ADC of total PUFA	99·2^b^	99·3^b^	99·3^b^	98·7^a^	0·1
ADC of total fatty acids	98·4^c^	98·3^c^	96·2^b^	90·2^a^	1·0

0_ScYE, 0 g/kg *Scizochytrium* sp. + yeast extract (control); 1_ScYE, 10 g/kg *Scizochytrium* sp. + yeast extract; 6_ScYE, 60 g/kg *Scizochytrium* sp. + yeast extract; 15_ScYE, 150 g/kg *Scizochytrium* sp. + yeast extract.

^a,b,c^ Mean values within a row with unlike superscript letters were significantly different (*P* < 0·05; one-way ANOVA as separated by Tukey's *post hoc* test).

* Heterotrophically produced *Scizochytrium* sp., spray dried and balanced to 500 g/kg total fat by yeast extract (Alltech Inc.).

### Microarray analyses

**Fig. 1.**
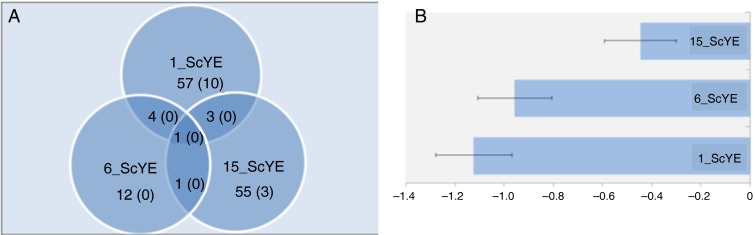
Microarray analyses in the liver of Atlantic salmon (*Salmo salar*) fed diets with graded ScYE (heterotrophically produced *Scizochytrium* sp., spray dried and balanced to 500 g/kg total fat by yeast extract) level (0, 1, 6 and 15 %). (A) The numbers of differentially expressed genes detected with microarrays. Genes were selected at signifcance thresholds *P* < 0·05 and *P* < 0·01 (values in parentheses). (B) Gene markers of erythrocytes. Data are mean log_2_-expression ratios with their standard errors for twelve genes: carbonic anhydrase, ammonium transporter RhB, erythroid-specific 5-aminolevulinate synthase (three features), haemoglobins (seven features).

The transcriptomic changes caused by the test diets were small as witnessed by the numbers of differentially expressed genes (Fig. 1(A)). A few genes passed the selection threshold in two groups and only one gene with unknown functions was differentially expressed in all groups. Nonetheless, a suite of genes showed either similar profiles across all groups or dose responses; examples are in Table 7. *p53* (up-regulated) plays a key part in the control of cellular growth and apoptosis, while *sesn1* (down-regulated) stimulates growth arrest in response to diverse stressors. *Sh3bgrl3* is involved in protection against oxidative stress and *abcg2a* expels toxic compounds from cells. Up-regulated *ppdpf* controls differentiation of exocrine cells. Only one gene (*mlyc,d*) takes part in the biosynthesis of fatty acids^(^^61^^)^. Common profiles were shown by erythrocyte-specific genes: their expression decreased at low levels of ScYE, while at high levels differences from control were minor (Fig. 1(B)).

**Table 7.** Examples of differentially expressed genes (microarray analyses) in the liver of Atlantic salmon (*Salmo salar*) fed diets with graded ScYE† inclusion levels (1, 6 and 15 %)‡

Gene	Function	1_ScYE	6_ScYE	15_ScYE
p53	Cell cycle, apoptosis	2·0	3·5*	2·5*
Myristoylated alanine-rich C kinase-like protein	Cell cytoskeleton	−1·5*	−1·4	−2·1*
Keratin, type I cytoskeletal 50 kDa	Cell cytoskeleton	3·5*	3·3	2·7
Centrosome-associated protein CEP250	Cell cytoskeleton	−2·7*	−1·9*	−1·1
SH3 domain-binding glutamic acid-rich-like protein 3	Cell redox	−1·1	−1·9	−2·6*
RNA binding motif protein 12	Cell RNA	1·8	1·6	3·6*
Synaptic vesicle 2-related protein	Cell secretion	2·4*	1·0	2·2
Rap guanine exchange factor	Cell signalling	2·7*	1·9	2·1
cGMP 3,5-cyclic phosphodiesterase subunit δ	Cell signalling	1·7	1·0	2·0*
Small ubiquitin-related modifier 2 precursor	Cell signalling	−1·7*	−2·1	−1·8*
Sestrin	Cell stress	−5·3	−3·0*	−1·9
Diencephalon/mesencephalon homeobox protein 1-A	Cell transcription	1·8*	2·2	1·5
Helicase-like transcription factor	Cell transcription	−1·9*	−1·7*	−2·3
Pancreatic progenitor cell differentiation and proliferation factor	Differentiation	1·1	1·7	2·1*
Urokinase-type plasminogen activator	Extracellular	1·0	−1·8	−2·3*
Collagen 6A3	Extracellular	−1·9	−1·7	−2·5*
Complement factor H1	Immune complement	−1·8*	−1·4	−1·6*
IL-16	Immune cytokine	−2·2	1·0	−1·8*
Lectin precursor	Immune lectin	−3·6*	−2·4*	−1·4
Glutamyl-tRNA(Gln) amidotransferase A, mitochondrial	Metabolism amino acids	−2·9*	−1·6	−2·2
l-Asparaginase	Metabolism amino acids	1·7*	2·8*	2·8*
Multiple inositol polyphosphate histidine phosphatase 1	Metabolism inositol	−1·8*	−1·1	−2·2*
Sodium/potassium/calcium exchanger	Metabolism ion	−1·3*	−2·3*	−2·0*
Chloride intracellular channel protein 2	Metabolism ion	−2·1*	−1·5*	−1·8
Malonyl-CoA decarboxylase, mitochondrial	Metabolism lipid	−1·9*	−1·8	−1·8*
Thymus-specific serine protease	Metabolism protease	−2·3*	−1·8	−1·3
ATP-binding cassette G2	Metabolism xenobiotic	−2·0*	−1·5*	−1·8
NACHT domain containing	Unknown	2·3*	1·9*	2·1*

1_ScYE, 10 g/kg *Scizochytrium* sp. + yeast extract; 6_ScYE, 60 g/kg *Scizochytrium* sp. + yeast extract; 15_ScYE, 150 g/kg *Scizochytrium* sp. + yeast extract; 0_ScYE, 0 g/kg *Scizochytrium* sp. + yeast extract (control); NACHT, NAIP (neuronal apoptosis inhibitory protein), CIITA (MHC class II transcription activator), HET-E (incompatibility locus protein from *Podospora anserina*) and TP1 (telomerase-associated protein).

* Significantly different values (*P* < 0·05).

† Heterotrophically produced *Scizochytrium* sp., spray dried and balanced to 500 g/kg total fat by yeast extract (Alltech Inc.).

‡ Data are fold changes compared with the control treatment (0_ScYE).

### Microscopic analysis of intestinal tissue and plasma creatine kinase

**Fig. 2.**
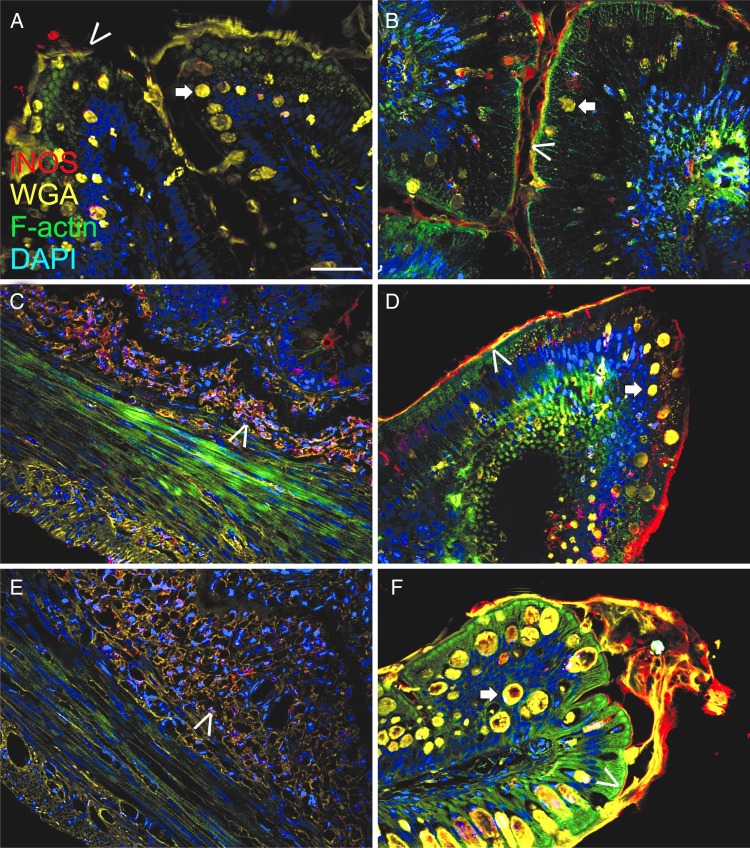
Immunofluorescence analysis of inducible nitric oxide synthase (iNOS) activity (pseudocoloured red) and fluorescence stainiong of F-actin (pseudocoloured green) and goblet cells (pseudocoloured yellow) in Atlantic salmon (*Salmo salar*) intestines fed variable levels of ScYE (heterotrophically produced *Scizochytrium* sp., spray dried and balanced to 500 g/kg total fat by yeast extract). Nuclei were stained with DAPI (4′,6-diamidino-2-phenylindole; pseudocoloured blue). (A) The control fish showed normal intestinal morphology and a low iNOS activity. Distribution and number of goblet cells (arrow) and apical F-actin (arrow head) in the enterocytes appeared normal. (B) Intestines from fish fed a diet with 1 % ScYE showed a normal morphology with respect to the number of goblet cells (arrow) and apical F-actin (arrow head), but slightly more intense apical iNOS activity in the enterocytes. (C) In fish fed 6 % ScYE we observed an increased iNOS activity in the submucosa (arrow head). (D) In the villus of fish fed 6 % ScYE apical iNOS was more intense (arrow head) and some enterocytes and goblet cells also showed increased iNOS staining. (E) Inclusion levels of 15 % ScYE revealed similar iNOS activity in the submucosa (arrow head), but the villus (F) appeared quite different from the control fish with increased number of swollen goblet cells (arrow), more intense F-actin staining (arrow head) and higher iNOS activity. White bar in Fig. 2(A) = 20 μm. WGA, wheat germ agglutinin.

Following histological examination of each feeding treatment (*n* 5), ScYE supplementation up to 15 % in the diet did not induce enteritis or abnormal intestinal morphology. However, an increasing dietary level of ScYE induced an increased number of slime cells as well as an increased oxidative stress response in the intestine of Atlantic salmon (Fig 2(A)–(F)). The control fish showed normal intestinal morphology and goblet cell numbers, as shown stained by wheat germ agglutinin (WGA), and modest levels of iNOS activity (Fig. 2(A): arrow head). Dietary inclusion of 1 % ScYE increased iNOS activity moderately (Fig. 2(B): arrow head) and there appeared to be somewhat more goblet cells (Fig. 2(B): arrow). In fish fed 6 % ScYE in the diet, up-regulation of iNOS was evident at the base of the intestine (Fig. 2(C): arrow head), whereas in the control fish, no iNOS was detected in this part of the intestine (not shown). Moreover, increased iNOS activity and more goblet cells were evident in the villi from fish fed 6 % ScYE in the diet (Fig. 2(D): arrow). Dietary inclusion of 15 % ScYE showed similar iNOS activity as for 6 % at the base of the intestine (Fig. 2(E): arrow head). In the villi a considerable increase in mucus, goblet cell number (Fig. 2(F): arrow) and iNOS activity was evident when 15 % ScYE was included in the diet (Fig. 2(F): arrow head). The plasma CK level showed no significant differences between the dietary treatments, but the plasma CK value in salmon from one of the tanks fed the 15_ScYE diet was severely elevated (246 220 U/l) compared with the average level. Hence, CK seems to a parameter that might be valuable to monitor in future studies.

## Discussion

Atlantic salmon performance in the present trial fed either fish oil or microalgae (ScYE) as an *n*-3 LC-PUFA supplement in the diet was optimal in all treatments as compared with previously reported trials using similar diets to the control of the present trial and similar fish sizes^(^^62^^,^^63^^)^ or other diets containing *Schizochytrium* sp. oil^(^^64^^)^. Fish in the 1_ScYE treatment with the highest postprandial blood plasma leptin levels than all treatments also had the highest growth, significantly when compared with fish in 15_ScYE, whereas the opposite may have been expected in a group with high feed intake rates and energy status. Except for 1_ScYE, among the fish in the other treatments postprandial plasma leptin correlated negatively with growth rather than feed intake, whereas in all treatments feed intake was positively correlating with FCR and not growth. This provides an indication that postprandial leptin blood plasma levels may reflect the growth status or potential of the fish fed diets with different levels of digestible lipid and energy^(^^65^^,^^66^^)^. Sissener *et al*.^(^^67^^)^ found no increase in leptin gene expression in fish fed high-plant protein and high-plant oil diets. Accordingly in our trial there was no difference in plasma leptin level of fish fed diet 15_ScYE which contained no fish oil and higher levels of rapeseed oil compared with the control and diet 6_ScYE.

In terms of dietary nutrient digestibility in salmon, the observed significant reduction in total lipid and gross energy ADC at the two highest dietary ScYE supplementation levels can be explained by the lower digestibility of the dietary SFA. This can be due to the limited capacity of Atlantic salmon to efficiently digest SFA at low temperatures and increasing dietary levels, as already reported by other studies^(^^68^^,^^69^^)^. Menoyo *et al*.^(^^69^^)^ found that fish oil SFA digestibility in salmon decreases at dietary levels above 13·5 % down to ADC values of 78 % at 32 % SFA levels in the dietary oil. 16 : 0 is the main SFA both in fish oil and ScYE showing decreasing ADC when fed to salmon at dietary levels above 10 %. SFA digestibility may also be affected by the positioning of the SFA in the microalgal TAG as previously suggested by Bracco^(^^70^^)^. Most fatty acids in ScYE are present in the form of TAG, the composition of which is currently unknown. Another hypothesis explaining reduced digestibility of microalgae nutrients is the integrity of cell walls, which may not be efficiently disrupted during processing and feed production or during digestion in the organism's intestinal tract. Nevertheless, following the fatty acid ADC results of the present study, showing very high PUFA and at the same time low SFA digestibility in the ScYE supplemented diets, this hypothesis seems improbable. The present results thus suggest that the extrusion method used to produce the algae-containing feeds is adequate processing for optimal cell wall disruption of these algae.

Though intestinal morphology of Atlantic salmon fed increasing levels of ScYE was unaffected, ScYE apparently led to innate immune responses, such as increased mucus production and number of Goblet cells, as recently also reported in other farmed fish species fed different microalgae products in the diet^(^^40^^,^^41^^)^. The innate immune system of fish is highly advanced^(^^71^^)^ and in some respects may be superior to the mammalian^(^^72^^,^^73^^)^, providing higher responses to induced stimuli. It is well known that mucus is an important physical barrier against intestinal bacteria; hence, our observations could point towards an improved gut health by dietary ScYE supplementation. However, increased mucus production is also observed as a response to intestinal irritation. Recently, Schreiber *et al*.^(^^74^^)^ showed that iNOS controls baseline mucus production and that disruption of the intestinal mucus layer due to inflammatory diseases such as bowel or Crohn's disease are followed by increased iNOs activity and mucus production. The observed intestinal activity of mucus and iNOS activity indicate that ScYE may affect intestinal health. However, we did not observe signs of enteritis even after 12 weeks and the growth performance of the fish suggests that the currently tested inclusion levels are acceptable to the fish.

The DHA incorporation in the salmon fillet of the present study was effective also in the absence of supplemental dietary fish oil in treatment 15_ScYE. According to the Global Organization for EPA and DHA Omega-3 (GOED), the consumer needs to be informed of the beneficial effects obtained with a daily DHA intake of 250 mg or 1·75 g per week. These levels of DHA intake can be met by eating approximately one portion of salmon (125 g) from the present experiments per week, namely 140 g fish fillet of the control, or 129 g fish fillet of the 0_ScYE, or 114 g fish fillet of the 6_ScYE, or 136 g fish fillet of the 15_ScYE treatment. Xu *et al*.^(^^75^^)^ found recently that Δ6 fatty acyl desaturase (*FADS2*) activity gene expression as well as the tissue levels of LC *n*-3 highly unsaturated fatty acids (HUFA) in the Japanese sea bass (*Lateolabrax japonicus*) were higher in fish fed palmitic acid-rich diets, in the form of tripalmitin, TAG:(16 : 0/16 : 0/16 : 0), compared with diets rich in either one of LC *n*-3 HUFA, stearic, oleic and α-linolenic acid. In the present study, the fish fillets of salmon fed diet 15_ScYE, containing the highest amounts of 16 : 0, no supplemental fish oil and low EPA levels, maintained the original levels of EPA. Though no mass balance analyses were performed, this fact indicates that farmed salmon smolt may efficiently preserve EPA in the tissues when this is not provided in excess by the diet^(^^76^^)^. This can be due to reduced β-oxidation of EPA and increased desaturation and elongation of shorter-chain fatty acids provided by the dietary plant oils or even by retro-conversion of the algal DHA to EPA. The fish at the end of the feeding trial were 4–5 times smaller than market-size fish when total fillet lipids are expected to be more than double than in the present study^(^^77^^)^ and so are expected to be the total amounts of DHA and total *n*-3 LC-PUFA. Thus, in terms of health and market standards, the experimental diets were well on the safe side of *n*-3 LC-PUFA levels, and in practice lower levels of algae biomass will be required in aqua feeds, allowing for lower feed cost formulations.

Liquid losses during storage are currently an issue in certain salmon fillet products, probably caused by the high level of unsaturated fatty acids present in the alternative plant oil sources used in fish feeds, such as rapeseed oil, causing both technical problems and nutrient wastage. In our trial, the fillet quality was equally good in all treatments in terms of gaping, texture and liquid losses during thawing despite the high rapeseed oil levels present in the 15_ScYE treatment. This may be explained by the maintenance of similar or higher levels of SFA in the fillet of the fish fed the ScYE-supplemented diets compared with the control, as ScYE is very rich in SFA and balances the very low saturation profile of supplemental rapeseed oil.

High-throughput gene expression profiling was performed to assess potential adverse dietary effects of microalgae. The microarray platform provides comprehensive coverage of diverse pathways and functional groups (online Supplementary Table S2) and taking into account results produced by our team in diverse studies, any signs of stress, toxicity, inflammation and other damage would be seen without a doubt. Overall, gene expression changes were small, being close to the margin of detectability, as also previously observed in gilthead sea bream fed microalgae in the diet^(^^78^^)^. We did not find enrichment of any pathway or functional category except for the markers of erythrocytes that showed slight reduction at the lowest level of algae. In the present study, we found only one regulated group including seventeen functionally related genes involved in overall oxygen transport and more specifically metabolism and transport of Fe and haeme (Table 7). Lower abundance of transcripts for 5-aminolevulinate synthase, the key enzyme of haeme biosynthesis, globins and Rhesus blood group-associated glycoprotein could imply that livers from salmon fed with test diets contained fewer erythrocytes. At the same time, the extracellular Fe transporter (transferrin) and the key endocrine regulator of Fe metabolism (hepcidin) were up-regulated with increasing levels of algae component, while the intracellular Fe storage protein (ferritin) showed the opposite regulation. The gene expression profiles most probably reflect a slight reduction in either blood circulation or erythropoiesis. However, this apparently had a minor impact on the fish. There were no signs of toxicity, stress, inflammation or any other negative effects from feeds with algae. Concerning lipid metabolism, only one enzyme (acyl-CoA synthetase long-chain family member 4a) showed higher expression in the 1_ScYE group. Together with minor overlap between the study groups, the observed expression changes most probably were sporadic in character. Several genes can be of interest if their differential expression is confirmed in further studies. To conclude, the microarray data support safety of the ScYE product for Atlantic salmon.

Overall, it is evident from the results of this trial that whole *Schizochytrium* sp.-based microalgae raw materials can provide both a good alternative to the depleting marine resources of *n*-3 LC-PUFA, and a realistic one, as this species can be produced efficiently using advanced fermentation technology in all parts of the world.

## Supplementary material

To view supplementary material for this article, please visit http://dx.doi.org/10.1017/jns.2015.14
